# Machine-Learning-Based Diagnostics of Cardiac Sarcoidosis Using Multi-Chamber Wall Motion Analyses

**DOI:** 10.3390/diagnostics13142426

**Published:** 2023-07-20

**Authors:** Jan Eckstein, Negin Moghadasi, Hermann Körperich, Rehsan Akkuzu, Vanessa Sciacca, Christian Sohns, Philipp Sommer, Julian Berg, Jerzy Paluszkiewicz, Wolfgang Burchert, Misagh Piran

**Affiliations:** 1 Institute for Radiology, Nuclear Medicine and Molecular Imaging, Heart and Diabetes Center North Rhine Westphalia, Bad Oeynhausen, University of Bochum, 32545 Bochum, Germany; jeckstein@hdz-nrw.de (J.E.);; 2 Department of Engineering Systems & Environment, University of Virginia, Charlottesville, VA 22904, USA; 3 Clinic for Electrophysiology, Heart and Diabetes Center North-Rhine Westphalia, Ruhr-University of Bochum, 32545 Bad Oeynhausen, Germany; vsciacca@hdz-nrw.de (V.S.);; 4 Clinic for Thoracic and Cardiovascular Surgery, Heart and Diabetes Center North-Rhine Westphalia, Ruhr-University of Bochum, 32545 Bad Oeynhausen, Germany; 5 Cardiology Institute and Clinic, Poznan University of Medical Sciences, 61-701 Poznan, Poland

**Keywords:** cardiac magnetic resonance, machine learning 2, cardiac sarcoidosis 3, cardiac strain, multi-chamber analyses

## Abstract

Background: Hindered by its unspecific clinical and phenotypical presentation, cardiac sarcoidosis (CS) remains a challenging diagnosis. Objective: Utilizing cardiac magnetic resonance imaging (CMR), we acquired multi-chamber volumetrics and strain feature tracking for a support vector machine learning (SVM)-based diagnostic approach to CS. Method: Forty-five CMR-negative (CMR(−), 56.5(53.0;63.0)years), eighteen CMR-positive (CMR(+), 64.0(57.8;67.0)years) sarcoidosis patients and forty-four controls (CTRL, 56.5(53.0;63.0)years)) underwent CMR examination. Cardiac parameters were processed using the classifiers of logistic regression, KNN(K-nearest-neighbor), DT (decision tree), RF (random forest), SVM, GBoost, XGBoost, Voting and feature selection. Results: In a three-cluster analysis of CTRL versus vs. CMR(+) vs. CMR(−), RF and Voting classifier yielded the highest prediction rates (81.82%). The two-cluster analysis of CTRL vs. all sarcoidosis (All Sarc.) yielded high prediction rates with the classifiers logistic regression, RF and SVM (96.97%), and low prediction rates for the analysis of CMR(+) vs. CMR(−), which were augmented using feature selection with logistic regression (89.47%). Conclusion: Multi-chamber cardiac function and strain-based supervised machine learning provides a non-contrast approach to accurately differentiate between healthy individuals and sarcoidosis patients. Feature selection overcomes the algorithmically challenging discrimination between CMR(+) and CMR(−) patients, yielding high accuracy predictions. The study findings imply higher prevalence of cardiac involvement than previously anticipated, which may impact clinical disease management.

## 1. Introduction

Sarcoidosis is a systemic, non-caseating granulomatous disease of unclear etiology affecting multiple organs [1]. The presentation of cardiac sarcoidosis ranges from asymptomatic to palpitations, syncope, symptoms of congestive heart failure and sudden cardiac death [2]. Although cardiac sarcoidosis (CS) is considered rare with symptomatic involvement estimated at 5% [3], it remains a detrimental predictor of mortality [4]. Moreover, autopsy results revealed that the majority of CS remains unrecognized, with actual cardiac involvement detected in approximately 27% of sarcoidosis patients [5]. Additionally, delayed immunosuppressive therapy decreased CS transplant-free survival from 83% to 53% at 10 years if heart failure was present at clinical presentation [6]. Hence, CS is often considered a clinical chameleon, further exemplified by the observation that the ECG is normal in 25% of CS patients despite extensive cardiac involvement [7]. Moreover, due to the patchy nature of myocardial involvement, endomyocardial biopsies yield an insufficient diagnostic rate [8]. Along with CMR(+)-associated congestive heart failure and conduction abnormalities, sudden death is one of the most common causes of mortality [9]. Therefore, methods for earlier and improved detection rate are essential.

Cardiac magnetic resonance (CMR) imaging is an effective diagnostic tool for CS detection [10], establishing diagnostic superiority over echocardiography [11]. Its diagnostic accuracy was underlined by a two-fold higher rate of CS detection with CMR compared to the standard guideline-based clinical evaluation [12]. In particular, late gadolinium enhancement (LGE) pattern is of high diagnostic and prognostic value and increases the odds of all-cause mortality and arrhythmogenic events for CS [13]. Nonetheless, the recent literature presents worrisome inconsistency among observers (low interobserver agreement) of LGE imaging, even at experienced centers, underscoring its limitation in CS detection [11,14]. Despite state-of-the-art cardiomyopathy protocols incorporating cardiac cine imaging, LGE and tissue characterization sequences, such as native and post-contrast T1-mapping, extracellular volume (ECV) quantification and T2-mapping [10], the CS detection rate appears to require improvement beyond the diagnostic performance of these features.

Cardiac wall deformation characterized by strain has been described as a promising diagnostic feature for the purpose of CS detection. Recent echocardiographic studies have demonstrated diagnostic discrimination based on cardiac functional and strain features between sarcoidosis patients and controls [15,16]. Moreover, CMR-quantified strain identified a potential early diagnostic marker of impaired left ventricular global longitudinal strain of sarcoidosis patients with otherwise normal CMR findings [17]. With the objective of optimizing CMR diagnostic accuracy, we hypothesize that multi-chamber-derived cardiac strain and function yield accurate diagnostic performance for CS. Analyses of the CMR-attained broad spectrum of cardiac features demands algorithmic assistance in the form of machine learning. Machine learning is a function of artificial intelligence and performs complex classification, regression and prediction of data matrices extending beyond the capacity of traditional prediction approaches. For example, a recently conducted study with similar design demonstrated competitive diagnostic accuracies for amyloidotic cardiomyopathy detection achieved via supervised machine learning [18]. Additionally, strain- and cardiac-function-based diagnostics offer novel perspectives for non-contrast CMR examination, which is relevant for sarcoidosis patients as hypercalcemia-induced renal dysfunction is not uncommon [19,20].

## 2. Methods

### 2.1. Study Cohort

This is a retrospective observational study, conducted at a single center. The study cohort consisted of 63 systemic sarcoidosis patients in whom the diagnosis was histologically validated from various tissues, including skin, lung or lymph nodes. The control group (CTRL) consisted of healthy volunteers. All volunteers submitted written informed consent prior to CMR examination. CTRL subjects were excluded if they had a medical history of cardiovascular disease, medication or surgery for cardiovascular disease, cardiovascular risk factors and metabolic disorders. All enrolled subjects received CMR at our center for evaluation of cardiac involvement. In patients with biopsy-proven extracardiac sarcoidosis, CMR was conducted on the basis of the Heart Rhythm Society diagnostic criteria [21] upon clinical suspicion of cardiac involvement. In adherence to guidelines of the Heart Rhythm Society (HRS) 2017 [22] recommending CMR for CS diagnostics, CMR findings were typically based on contrast-enhanced myocardial scarring patterns, myocardial edema, quantitative tissue characterization and cardiac functional analysis. Patients were classified as CMR-positive (CMR(+)) if their CMR examination demonstrated CS-associated features, or CMR-negative (CMR(−)) if no signs of cardiac involvement were found. All enrolled sarcoidosis patients received routine CMR according to standard cardiomyopathy protocol with adequate imaging quality. Pathological CMR findings, such as ischemic or non-ischemic heart disease and vascular abnormalities, would further result in exclusion of the volunteer from the study. All examinations were conducted in accordance with the 1964 declaration of Helsinki and the approval of the local ethics committee was waived (Ethik-Kommission der Medizinischen Fakultät der Ruhr-Universität Bochum; registration number 2023-1071).

### 2.2. Cardiac Magnetic Resonance Imaging

The CMR imaging was performed using a 3.0 Tesla multi-transmit magnetic resonance scanner (Achieva, Philips Healthcare, Best, The Netherlands; Release 5.3.1 and 5.6.1) incorporating dStream technology. All patients underwent vector electrocardiogram-triggered cardiac acquisitions. The maximum gradient performance was 40 mT/m with a slew rate of 200 mT/m/ms. A cardiac phased-array coil was used for signal reception. An axially acquired stack covering the whole heart and a short-axis stack covering the entire left and right ventricles (12–16 slices, no gap) as well as standard two-, three- and four-chamber views were utilized with retrospectively gated cine steady-state free-precession acquisitions (TR/TE/flip angle = 2.7 ms/1.35 ms/42°) for the assessment of heart function in all four cardiac chambers and morphology. Parallel imaging technique with a SENSE-reduction factor of 2 was applied to keep breath-holding times ≤12 s. Within one cardiac cycle, >25 reconstructed heart frames were acquired in order to achieve greater temporal resolution, as recently demonstrated [23]. The spatial resolution was 1.5 × 1.5 × 8 mm^3^.

### 2.3. Cardiac Function and Strain Quantification

For cardiac function and strain analysis on cine steady-state free-precession acquisitions, CVI42^®^ software package (Circle Cardiovascular Imaging Inc., Calgary, AB, Canada, Version: Release 5.16.2 (3381)) was utilized (for parameter list see Table 1). Left ventricular (LV) and left atrial (LA) endocardial and epicardial contouring was performed in longitudinal 2-chamber and 4-chamber views. LA contouring excluded the left atrial appendage and ostia of the pulmonary veins. Only the 4-chamber view was employed for analysis of the right atrium (RA). RA contouring excluded the right atrial appendage and superior and inferior ostia of the vena cava. Automatic volumetric and strain analysis for the LV and right ventricle (RV) contouring additionally entailed the short-axis stacks. Manual adaption was performed if needed. Volumetric biventricular and biatrial quantification were obtained using the disc-summation technique (Simpson approach) at end-diastole and end-systole.

### 2.4. Descriptive Statistics

Descriptive statistics were generated using SPSS (version 27.0.0.0, IBM Deutschland GmbH, iBM, Armonk, NY, USA). For normally distributed continuous variables, the mean ± standard deviation was reported, whereas for non-parametric variables, the median with interquartile range was provided. For comparison of baseline characteristics, either the univariate analysis of variance (ANOVA) or the Welch test were employed if the assumptions of ANOVA were not met. For determining inter-group differences, a post hoc Tukey’s Honest Significant Difference (HSD) test was conducted when there was homogeneity of variance. If homogeneity of variance was violated, a Games–Howell test was used. In the case of nonparametric data, the Kruskal–Wallis test was applied.

### 2.5. Cardiac Features

Multiparametric CMR assessment encompassed bi-atrial and bi-ventricular volumetric and functional parameters along with strain and strain rate of the left and right atrium and left ventricle, cumulatively making up 36 cardiac features.

### 2.6. Correlation Matrix

Linear correlation coefficients were derived using Spearman’s correlation between variables forming a matrix. The coefficients span from −1 to +1, where +1 demonstrates a strong positive correlation and vice versa. Normalization for all data was performed for bias reduction.

### 2.7. Classification Algorithms

The generated coefficients were fed into the following machine learning classifier algorithms: support vector machine (SVM), k-nearest-neighbor (KNN), decision tree (DT), random forest (RF), logistic regression, GBoost, XGBoost and Voting. Simplified descriptions along with illustrations of various algorithmic functions were recently described [18]. Mella and Pentakoti define the Voting classifier as a machine learning model that combines the predictions of multiple individual models to make a final prediction. Instead of using a single model, it creates an ensemble by training on numerous models. The Voting classifier predicts the output class based on the highest probability or majority vote from the individual models. By doing so, it simplifies the process of model selection and achieves better accuracy by leveraging the collective wisdom of the ensemble. Rather than training and evaluating separate models individually, a single model is created that makes predictions based on the combined majority voting of the ensemble’s models for each output class [24]. Data were randomly assigned into a training or testing category. The training set is 70% of the dataset used to train a model. It consists of input data along with corresponding known output values, allowing the model to learn from this labeled data. During training, the model adjusts its internal parameters and learns patterns from the training set to make accurate predictions.

**Figure 1:**
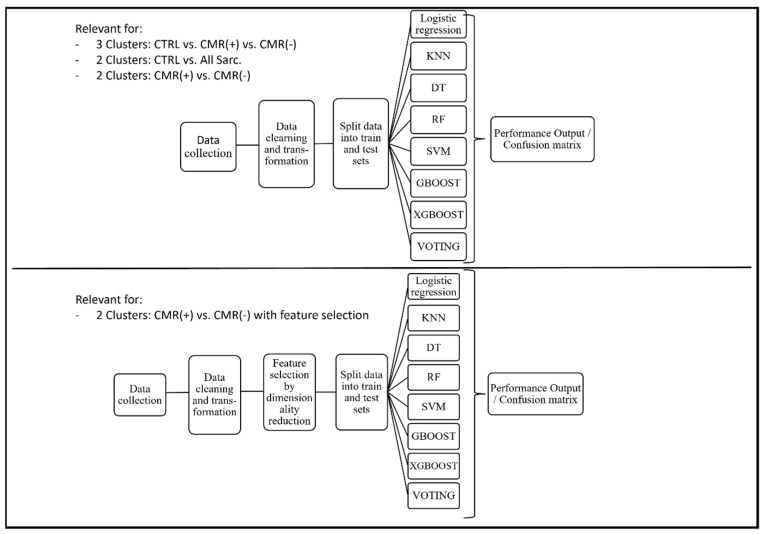
Flow chart illustrating the processing steps of machine learning along with the applied classifiers. CTRL—control subjects, CMR(+)—positive cardiac magnetic resonance patients, CMR(−)—negative cardiac magnetic resonance patients, KNN—K-nearest-neighbor, DT—deep trees, RF—random forest, SVM—support vector machine, GBOOST—gradient boosting, XGBOOST—extreme gradient boosting.

Once the model is trained, it is essential to assess its performance on new, unseen data. This is where the remaining 30% of the data come into play as the test set. The test set is a separate dataset that was not used during training. It contains input data, similar to the training set, but lacks the corresponding labels. The trained model then makes predictions on the test set, and its performance is evaluated by comparing these predictions to the true labels withheld from the model (Table 2). A flow chart of data processing is given by Figure 1.

SVM utilizes a hyperplane for discrimination between two clusters. Flexibility of the hyperplane affects accuracy. KNN orientates around the Euclidian distance, where similar variables remain in closer algorithmic proximity than dissimilar data. DT processing is algorithmically structured into “nodes” for variables and “branches” for decisions. Random forest is characterized as an extension of DT, whereby a multitude of decision trees may generate random forests, which can correct for overfitting and generally outperform DT [25]. As defined by Mohd Ali et al., random forest classifier is also a machine learning model used in both classification and regression problems that, unlike fitting a single “best” tree model, strategically combines multiple simple decision trees to optimize predictive performance. This approach accommodates diverse travel decision heuristics by utilizing a multitude of decision trees. Each tree in the ensemble captures different sources of uncertainty and variability in the data. Consequently, this technique enhances the accuracy of model estimation and prediction [26]. Lastly, logistic regression operates by estimating parameters using logistic function to convert log-odds into probability. A voting classifier is a machine learning model predicting output class based on the majority of votes from an ensemble of models. It supports two types of voting: hard voting, which takes the class with the highest majority of votes; and soft voting, which takes the class with the highest average probability. GBoost refers to gradient boosting classifier, which merges groups of weak learning models to augment their predictive performance, particularly via reweighting of data. XGBoost refers to extreme gradient boosting, commonly based on a DT model which applies customized algorithms to maximize the predictive potential. Due to poor prediction rates of CMR(+) vs. CMR(−) discrimination, feature selection was applied. Feature selection is important in machine learning to reduce data complexity and improve accuracy. It focuses on selecting relevant features, which improves model performance, prevents overfitting, enhances interpretability, reduces computational complexity and optimizes data collection and storage. This results in higher performance of the models to classify CMR(+) vs. CMR(−). All data processing was performed using Python (Version No. 3.8.12). Algorithmic differentiation requires identification of hyperparameters utilizing the support vector classifier, ultimately forming a “best fit” hyperplane of the dataset. Algorithmic fine-tuning was conducted throughout the training and testing of the individual classifiers. Output of the individual classifier algorithms is presented in confusion matrices and summarized in terms of precision (positive predictive value), recall score (sensitivity) and the F1-score (test accuracy).

### 2.8. Algorithmic Feature Selection

Algorithmic feature selection was applied to the analyses of CMR(+) vs. CMR(−) patients. The individual contributions of each feature were evaluated based on RF classifier and presented via feature rates. The five features with the highest predictive values were selected to augment diagnostic accuracy. A closer proximity towards “1” would imply that this feature positively predicts cardiac sarcoidosis. To validate performance enhancement using feature selection application, ROC analyses were carried out to compare discrimination between cluster CMR(+) vs. CMR(−) without and with feature selection.

## 3. Results

### 3.1. Baseline Features

This study enrolled 44 control subjects, 45 CMR(−) and 18 CMR(+) patients. CMR(+) (median (interquartile range), 64.0(57.8;67.0)years) patients represented the oldest patient group in contrast to CMR(−) (mean ± standard deviation, 56.2 ± 15.5 years) and CTRL (56.5(53.0;63.0)years), without statistical significant difference. The left (LV-EF) and right (RV-EF) ventricular ejection fraction (%) of both CMR(−) and CMR(+) patients was significantly reduced in contrast to CTRL (e.g., LV-EF: 49 ± 15; 61 (50;65); 67 ± 5; *p* < 0.001). CMR(+) patients demonstrated significantly elevated left ventricular volumetrics compared to CMR(−) patients and CTRL; for example, regarding indexed end-diastolic volume (96.5 ± 26.0; 69.3 (60.0;83.7); 71.6 ± 9.6; *p* < 0.001). In contrast to CTRL, left ventricular longitudinal, radial and circumferential strains were found to be significantly impaired for CMR(+)patients (*p*-values: <0.001–0.007). Further details are summarized in Table 1.

### 3.2. Diagnostic Accuracy of Machine Learning Models

**Figure 2:**
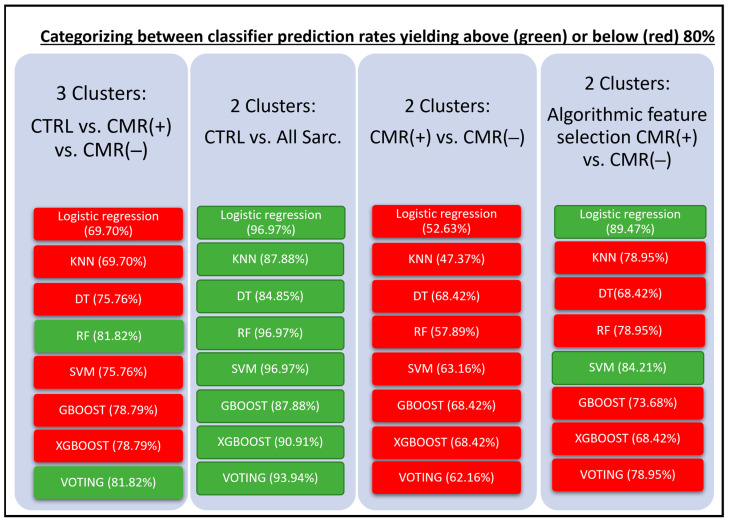
Graphic discrimination between classifier performance of the different analyses, yielding below (red) and above (green) 80% prediction rates with corresponding outcome statistics. CTRL—control subjects, CMR(+)—positive cardiac magnetic resonance patients, CMR(−)—negative cardiac magnetic resonance patients, All Sarc.—all sarcoidosis patients, KNN—K-nearest-neighbor, DT—deep trees, RF—random forest, SVM—support vector machine, GBOOST—gradient boosting, XGBOOST—extreme gradient boosting.

The CMR multi-chamber wall motion and functional analyses resulted in a 36-feature matrix for algorithmic processing (supplemental data Tables S1–S3). Among the various classifier algorithms used, RF and Voting classifier demonstrated the highest level of prediction rate for the differentiation between the three clusters of CTRL, CMR(−) and CMR(+) patients (82%). Analyses of CTRL versus All Sarc. found logistic regression, RF and SVM to yield the highest prediction rates (97%). Algorithmic discrimination between CMR(+) versus CMR(−) appeared challenging, reaching maximum accuracy of 68% for the DR, GBoost and XGBoost classifiers. Differentiation between CMR(+) versus (CMR(−) analyses was augmented using algorithmic feature selection, raising it to a maximum accuracy of 89% utilizing logistic regression. Further details are summarized in Figure 2.

### 3.3. Confusion Matrices

**Figure 3:**
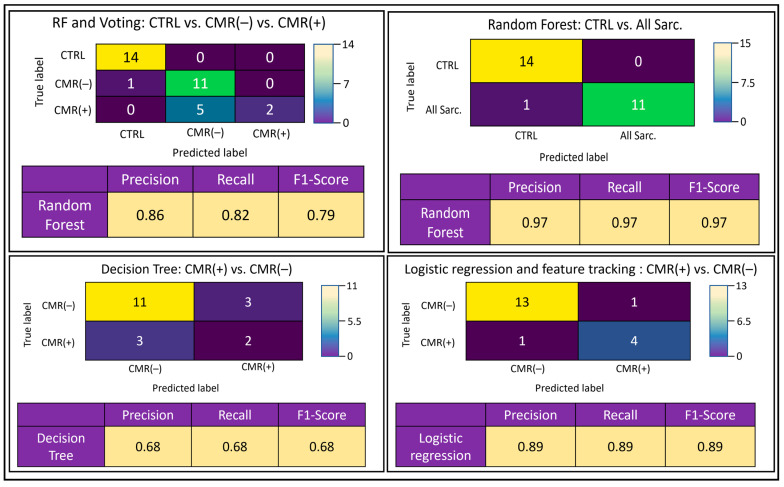
Selected confusion matrices for 36-feature analyses of the various cluster analyses, depicting the highest yielding classifier(s) per cluster with corresponding performance statistics. Precision—positive predictive value, recall score—sensitivity, F1-score—test accuracy, CTRL—control subjects, CMR(+)—positive cardiac magnetic resonance patients, CMR(−)—negative cardiac magnetic resonance patients, All Sarc.—all sarcoidosis patients, DT—deep trees, RF—random forest.

Confusion matrices were generated and exemplary top-performing classifier algorithms were exhibited (Figure 3). These presented differences between the true and predicted outcomes based on the testing data category. The confusion matrices demonstrate that CTRL subjects were predicted with highest accuracy in the three-cluster model; for example, based on a precision (positive predictive value) of 86%, recall (sensitivity) of 82% and F1-score (test accuracy) of 79%. In particular, differentiation of CTRL from All Sarc. in the two-cluster model appeared effective with regard to a constant 97% precision, recall and F1-score. However, inter-group differences for sarcoidosis patients upon division into CMR(+) and CMR(−) patients resulted in poor performance, with a constant 68% precision, recall and F1-score. Algorithmic accuracy was augmented using algorithmic feature selection, yielding 89% precision, recall and F1-score.

### 3.4. Algorithmic Feature Selection

**Figure 4:**
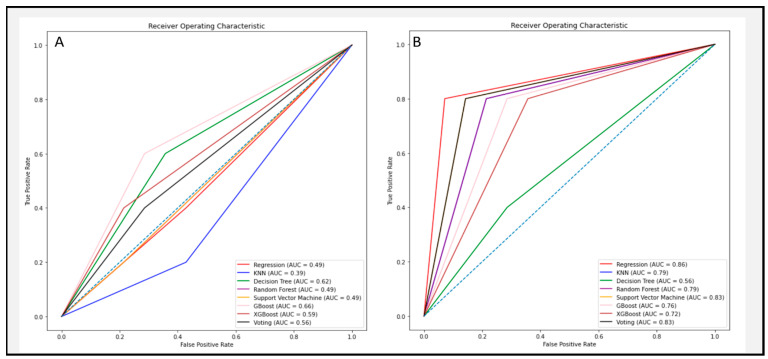
ROC curves for (**A**) CMR(+) vs. CMR(–) with no feature selection and (**B**) (+) v. CMR(–) after selecting features.

The five features with the highest algorithmic impact for the two-cluster model of CMR(+) versus CMR(−) are displayed in Table 2. All five parameters were based on left ventricular cardiac motion or volumetrics. In particular, longitudinal strain rates of the left ventricle appeared to be of high discriminative value for machine learning algorithms. Among the most valuable impact features for both analyses was routinely available indexed left ventricular end systolic volume. The ROC curves of Figure 4 demonstrate a profound enhancement of model performance in distinguishing the two clusters after selecting the most important features.

## 4. Discussion

To our knowledge, this study is the first to demonstrate diagnostic prediction rates for cardiac sarcoidosis based on CMR-acquired multi-chamber wall motion and volumetrics analyses using supervised machine learning algorithms.

The present study reports the following novelties:

(i) Accurate algorithmic discrimination was achieved between healthy subjects and all sarcoidosis patients, particularly with Voting and RF classifiers;
(ii) Poor algorithmic discrimination of CMR(−) and CMR(+) patients was improved to accurate levels via algorithmic feature selection application, particularly when using logistic regression and SVM classifiers;
(iii) The algorithmic challenge associated with discrimination between both patient groups implies cardiac involvement may be more prevalent than anticipated, potentially evading CMR detection.

### 4.1. The Benefit of Machine Learning in Cardiac Imaging

Implementations of machine learning algorithms in recent non-CS cardiac imaging have presented additive benefit for diagnostic support [27,28,29]. The present CMR study showed highly accurate discrimination of healthy subjects from all sarcoidosis patients. In terms of the confusion matrices, diagnostic prediction rate was higher for discrimination between healthy individuals and all sarcoidosis compared to distinguishing between CMR(−) and CMR(+) patients. A possible explanation may be the limited number of confirmed CMR(+) subjects included, which in the context of machine learning may result in imbalanced class distribution that can affect the accuracy of the model. Most interesting was the poor algorithmic discrimination between CMR(−) versus CMR(+) patients in the two-cluster model, for which promising algorithmic accuracy was expected given the baseline data. Based on the multi-parametric data input, it can be surmised that multi-chamber wall motion and volumetrics have a greater overlap than expected, compromising algorithmic discrimination. Utilizing feature selection application, accurate prediction rates between CMR(+) versus CMR(−) patients were maintained, based primarily on left ventricular wall motion features. Thus, machine learning is principally capable of distinguishing between both CMR groups. However, this study holds a greater serendipitous value, which implies that cardiac involvement tends to be a commonality rather than exception. This is further underscored by accurate algorithmic discrimination between CTRL and all sarcoidosis patients in the three- and two-cluster models. These findings indicate that cardiac involvement is more prevalent than recognized, perhaps at a subvisual level, in agreement with echocardiographic findings [16,30,31].

To the best of our knowledge, machine learning has not been applied to CMR data for the purpose of cardiac sarcoidosis prediction. A recent study by Kasushika et al. applied deep learning algorithms to echocardiographic movies for CS detection and found no significant difference between the area under the curve (AUC) of the pretrained algorithm in contrast to the diagnostic accuracy of five experienced cardiologists [32]. Moreover, they observed lowered AUC and negative predictive value compared to the cardiologists’ interpretation for CS with preserved LVEF, but did not reach statistical significance. An additional benefit of machine learning integration was presented when using a deep convolutional neural network (DCNN) applied to polar maps of F-FDG PET, identifying the greatest sensitivity and specificity for when using the ReliefF classifier for feature weight estimation, in contrast to standardized uptake value (SUV)-based classification and coefficient of variance (CoV)-based classification [33]. These examples underscore the benefit of implementing machine learning algorithms as a tool for diagnostic support. Nonetheless, machine learning application for cardiac imaging data is currently in its infancy, requiring larger cohort studies for validation.

### 4.2. Weighting of the Input Features

Various imaging studies have demonstrated that cardiac wall deformation is a valuable parameter for CS detection, capable of discriminating from healthy subjects [15,16,34]. In contrast to the focus of prior cardiac imaging studies [15,16,30,31], left ventricular strain rate rather than strain received greater diagnostic weighting for algorithmic discrimination. However, echocardiographic findings from Di Stefano et al. substantiate this observation, reporting compromised left ventricular longitudinal, circumferential, longitudinal early diastolic and circumferential early diastolic strain rates in CS patients with positive endomyocardial biopsy [34]. The power of machine learning lies in its ability to be adjustable, per example in the form of reweighting features. Shifting algorithmic weighting towards left ventricular wall motion features augmented discrimination between both CMR groups, emphasizing the adaptability of machine learning.

### 4.3. Clinical Outlooks

The mismatch between the relatively low clinical detection rate of CMR(+) in contrast to CMR(−) sarcoidosis patients implies that CS can remain undetected and that diagnostic methods require optimization. The recent literature has demonstrated that clinical data, such as from echocardiography and ECG, only reasonably predict positive CMR or FDG-cPET findings for the purpose of CS detection [35], reiterating its clinical chameleon-like nature. Baseline data have found single parameters, such as left ventricular strains or ejection fraction, to appear sufficient for diagnostic differentiation. Although these may represent useful diagnostic indicators, their entirety may not suffice for diagnostic manifestation. Instead, the present study assesses the potential of machine-learning-processed multi-parametric CMR data with the objective of pushing up the boundaries of diagnostic accuracy rates. Due to the rarity and complexity of the disease, generating a validated algorithm for CS diagnosis is challenging. This study presents novel diagnostic aspects of non-contrast CMR, for which machine learning of multi-chamber volumetrics and strain achieved high diagnostic discrimination between healthy subjects and sarcoidosis patients. This binary distinction represents a novel additive clinical benefit of machine learning integration, requiring validation in larger cohort studies. Moreover, whilst machine learning can be adjusted to distinguish between both CMR groups, this study found signs that cardiac involvement appears far more widespread than anticipated. If validated by future studies, these findings would alter clinical disease management, such as in patient monitoring and earlier therapeutic strategies.

## 5. Limitations

This study utilized a retrospective single-center observational design with typical limitations. Considering that this study was conducted at a specialized cardiovascular clinic, it is important to acknowledge the presence of selection bias in our cohort. This implies that the level of cardiac involvement observed among our participants is likely higher compared with other studies of patient cohorts with systemic cardiac sarcoidosis. Furthermore, with regard to the baseline data, we acknowledge that CMR(+) patients were, on average, detected in more advanced stages of the disease. Therefore, we cannot make claims regarding early disease detection based on these data. Although the cohorts were not matched, the study included healthy individuals over the age of 50 to improve data homogeneity. Moreover, it is important to note that overlapping structural changes associated with further underlying diseases, such as hypertensive heart disease, cannot be ruled out. The current study demonstrates a supervised machine learning approach. Its diagnostic applicability in an unsupervised setting has not been examined. Moreover, while algorithms can identify numerical patterns in large datasets and provide supportive output to physicians in clinical practice, they cannot establish a cause-and-effect relationship. It is essential to recognize that this study is preliminary in nature and requires further validation with larger datasets and additional risk assessment.

## 6. Conclusions

Supervised machine learning based on multi-chamber strains and volumetrics provides a non-contrast and non-invasive approach to effectively discriminate between healthy and sarcoidosis patients that is most competitive when using Voting and RF classifiers. Feature selection facilitates the otherwise difficult discrimination between CMR(+) and CMR(−) sarcoidosis patients, especially when using logistic regression and the SVM classifier. Our results suggest that cardiac involvement is more widespread than assumed and may evade CMR detection, potentially necessitating changes in clinical disease management.

## Figures and Tables

**Table 1:** Baseline data of control subjects and sarcoidosis patients.

	CRTL	CMR(−)	CMR(+)	Comparison	*p*-Values
**Number**	44	45	18		
**Male**	23	19	12		
**Age ^c^**	56.5(53.0;63.0) ^§^	56.5(53.0;63.0) ^§^	64.0(57.8;67.0) ^§^		n.s.
**Weight ^b^**	74.2 ± 12.5	81.9 ± 15.1	85.6 ± 17.0	CTRL—CMR(−) CRTL—CMR(+)	**0.036** **0.016**
**Height ^c^**	172 ± 10	168(164;177) ^§^	177 ± 10		n.s.
**Heart rate ^b^**	65 ± 10	72 ± 13	71 ± 13	CTRL—CMR(−)	**0.015**
**BMI ^c^**	24.8 ± 2.6	28.0 ± 4.5	27.3 ± 4.8	CTRL—CMR(−)	**<0.001**
**LV_EF ^c^**	67 ± 5	61(50;65)	49 ± 15	CTRL—CMR(−) CRTL—CMR(+)	**<0.001** **<0.001**
**LV_ESVi ^c^**	23.0(20.9;26.8) ^§^	27.9(21.1;38.3) ^§^	50.2 ± 21.2	CTRL—CMR(−) CRTL—CMR(+) CMR(−)—CMR(+)	**0.022** **<0.001** **0.004**
**LV_EDVi ^c^**	71.6 ± 9.6	69.3(60.0;83.7) ^§^	96.5 ± 26.0	CRTL—CMR(+) CMR(−)—CMR(+)	**<0.001** **<0.001**
**LV_longitudinal_strain ^c^**	−16.5 ± 2.4	−15.9(−17.5;−14.4) ^§^	−10.9 ± 3.8	CRTL—CMR(+) CMR(−)—CMR(+)	**<0.001** **0.001**
**LV_radial_strain ^b^**	29.5 ± 7.4	26.7 ± 8.5	19.9 ± 7.5	CRTL—CMR(+) CMR(−)—CMR(+)	**<0.001** **0.007**
**LV_circumferential_strain ^c^**	−17.3 ± 3.1	−17.1(−18.7;−15.2) ^§^	−12.8 ± 4.0	CRTL—CMR(+) CMR(−)—CMR(+)	**<0.001** **0.005**
**RV_EF ^c^**	61 ± 6	49.5(40.7;56.3) ^§^	44.1 (26.2;53.6) ^§^	CTRL—CMR(−) CRTL—CMR(+)	**<0.001** **<0.001**
**RV_ESVi ^c^**	30.1 ± 8.1	38.4(29.2;48.7) ^§^	44.4 (36.7;74.7) ^§^	CTRL—CMR(−) CRTL—CMR(+)	**0.003** **<0.001**
**RV_EDVi ^c^**	76.0 ± 12.7	71.1(63.7;88.5) ^§^	83.1 (66.1;92.2) ^§^		n.s.

^b^—ANOVA–Tukey–HSD, ^c^—Kruskal–Wallis test, (^§^)—median (interquartile range), n.s.—not significant, CTRL—control subjects, CMR(+)—CMR-positive patients, CMR(−)—CMR-negative patients. LV_EF—left ventricular ejection fraction, LV_ESVi—indexed left ventricular end systolic volume, LV_EDVi—indexed left ventricular end diastolic volume, LV_longitudinal_strain—left ventricular longitudinal strain, LV_radial_strain, LV_circumferential_strain, RV_EF—right ventricular ejection fraction, RV_ESVi—indexed right ventricular end systolic volume, RV_EDVi—indexed right ventricular end diastolic volume.

**Table 2:** Feature rates of the five most valuable parameters for machine learning discrimination generated by random forest classifier.

Parameter	Feature Rates
**LV_ESVi**	0.045
**LV_syst_radial_LAX_SR**	0.045
**LV_diast_radial_LAX_SR**	0.049
**LV_radial_LAX_S**	0.056
**LA_syst_long_LAX_SR**	0.060

LV_ESVi—indexed left ventricular end systolic volume, LA_syst_radial_LAX_SR—left atrial systolic radial strain rate in longitudinal axis, LA_diast_radial_LAX_SR—left atrial diastolic radial strain rate in longitudinal axis, LV_radial_LAX_S—left ventricular radial strain in longitudinal axis, LA_syst_long_LAX_SR—left atrial systolic longitudinal strain rate in longitudinal axis.

## Data Availability

Data is available on request due to restrictions for privacy protection.

## Supplementary Materials

The following supporting information can be downloaded at: https://www.mdpi.com/article/10.3390/diagnostics13142426/s1, Table S1: Data matrix derived from clusters CTRL, CMR(+) and CMR(−); Table S2: Data matrix derived from clusters CTRL and CS; Table S3: Data matrix derived from clusters CMR(+) and CMR(−).
