# Supplementary material for: Machine-Learning-Based Diagnostics of Cardiac Sarcoidosis Using Multi-Chamber Wall Motion Analyses
Source: Diagnostics (Basel). 2023 Jul 20;13(14):2426. doi: 10.3390/diagnostics13142426 (PMC10377893; doi:10.3390/diagnostics13142426)
Supplement: Supplementary file 1 [file diagnostics-13-02426-s001.zip › S2_CTRL_CS_matrix.pdf]

	LV_EDV	LV_ESV	LV_SV	LV_EF
LV_EDV	1	0.779395162	0.58244691	-0.406287501
LV_ESV	0.779395162	1	-0.055241418	-0.857145134
LV_SV	0.58244691	-0.055241418	1	0.463979421
LV_EF	-0.406287501	-0.857145134	0.463979421	1
LV_EDV/BSA	0.940779845	0.714440982	0.5722271	-0.357036542
LV_ESV/BSA	0.745043121	0.982542323	-0.086968266	-0.865392832
LV_radial_SAX_S	-0.414495026	-0.654705211	0.187580197	0.693545637
LV_circumf_SAX_S	0.451741037	0.711969411	-0.202685131	-0.725128443
LV_syst_radial_SAX_SR	-0.339315935	-0.407638551	-0.012284182	0.385447063
LV_syst_circumf_SAX_SR	0.237180602	0.235053821	0.072953657	-0.182295292
LV_diast_radial_SAX_SR	0.348822241	0.536384836	-0.139066273	-0.567106667
LV_diast_circumf_SAX_SR	-0.328779288	-0.507004999	0.13314694	0.511037117
LV_radial_LAX_S	-0.480328642	-0.636539399	0.060205218	0.569561085
LV_time_to_peak_long_LAX	0.238659968	0.137882747	0.201543854	-0.009142377
LV_syst_radial_LAX_SR	-0.419196758	-0.511269301	-0.010632692	0.473939457
LV_syst_long_LAX_SR	0.515770922	0.594330623	0.050843155	-0.504962527
LV_diast_radial_LAX_SR	0.370970729	0.533759561	-0.09886576	-0.495401729
LV_diast_long_LAX_SR	-0.379943328	-0.533422852	0.084077253	0.488164383
RV_EDV	0.678385389	0.477136995	0.462359359	-0.211355952
RV_ESV	0.65007194	0.641708333	0.203086138	-0.473287482
RV_SV	0.090761338	-0.267695967	0.493131657	0.462941502
RV_EF	-0.473823427	-0.668506272	0.113124079	0.706312552
RV_EDV/BSA	0.579400295	0.413861357	0.386611611	-0.179246129
RV_ESV/BSA	0.590694897	0.603759458	0.157621123	-0.465085129
LA_radial_LAX_S	0.382933904	0.396041338	0.09489941	-0.273855642
LA_long_LAX_S	-0.385458752	-0.427714188	-0.057655564	0.320668581
LA_syst_radial_LAX_SR	0.143551038	0.213126523	-0.048428666	-0.254458203
LA_syst_long_LAX_SR	-0.292219129	-0.402167254	0.057286865	0.434243624
LA_diast_radial_LAX_SR	-0.304513329	-0.344136087	-0.03914468	0.262147069
LA_diast_long_LAX_SR	0.461225419	0.417783906	0.191905718	-0.279568213
RA_radial_LAX_S	0.454876882	0.343086091	0.28023783	-0.213546913
RA_long_LAX_S	-0.323849634	-0.313636533	-0.110700433	0.204222107
RA_syst_radial_LAX_SR	0.316419532	0.296206083	0.120307728	-0.28698655
RA_syst_long_LAX_SR	-0.280204917	-0.285388537	-0.077719229	0.23920599
RA_diast_radial_LAX_SR	-0.464287962	-0.348415211	-0.288228642	0.18577694
RA_diast_long_LAX_SR	0.416398561	0.280404686	0.300795974	-0.107521548
Cardiac Sarcoidosis	0.263812476	0.373286581	-0.062769007	-0.432986577

LV_EDV/BSA	LV_ESV/BSA	LV_radial_SAX_S	LV_circumf_SAX_S	LV_syst_radial_SAX_SR
0.940779845	0.745043121	-0.414495026	0.451741037	-0.339315935
0.714440982	0.982542323	-0.654705211	0.711969411	-0.407638551
0.5722271	-0.086968266	0.187580197	-0.202685131	-0.012284182
-0.357036542	-0.865392832	0.693545637	-0.725128443	0.385447063
1	0.737574037	-0.373460728	0.413373028	-0.272681402
0.737574037	1	-0.6640571	0.71871335	-0.393784549
-0.373460728	-0.6640571	1	-0.967403298	0.537848026
0.413373028	0.71871335	-0.967403298	1	-0.513476879
-0.272681402	-0.393784549	0.537848026	-0.513476879	1
0.180294643	0.219477957	-0.26479962	0.249924136	-0.883006913
0.276233665	0.525193834	-0.769475333	0.739532397	-0.4287553
-0.271908162	-0.499586109	0.651410159	-0.644138993	0.361244035
-0.430064545	-0.630930239	0.693731489	-0.73391632	0.374737899
0.263628613	0.136314368	-0.023402323	0.035284881	-0.08249272
-0.348598217	-0.505092256	0.477343727	-0.454253828	0.307578693
0.424044453	0.567576051	-0.514834079	0.511510582	-0.366873049
0.276892973	0.514409874	-0.563373617	0.573654106	-0.349052545
-0.280056018	-0.514369861	0.509486416	-0.507957878	0.317587311
0.559436378	0.420659116	-0.183814616	0.175124846	-0.210575052
0.590541215	0.621885887	-0.367249334	0.377753592	-0.217933224
-0.033796757	-0.341112995	0.322598864	-0.35865641	-0.00413453
-0.457732418	-0.687341061	0.54345803	-0.553300257	0.245000688
0.558632497	0.408942789	-0.152764825	0.143279145	-0.150446853
0.567806977	0.605920205	-0.356853655	0.360510443	-0.186242752
0.417223621	0.404573679	-0.283654119	0.338545403	-0.124889199
-0.37567402	-0.422040948	0.372535947	-0.413051832	0.20280384
0.15535754	0.219874691	-0.139116041	0.113902686	-0.038823093
-0.277224692	-0.402029869	0.269947824	-0.287036935	0.167974108
-0.326291053	-0.357486224	0.211335935	-0.259465874	0.125944379
0.46359118	0.416073571	-0.372387995	0.398024929	-0.259096338
0.423650661	0.301804879	-0.233386227	0.23525372	-0.171985375
-0.254622791	-0.269530503	0.345760586	-0.342487875	0.180275223
0.344788347	0.305164712	-0.250674219	0.270093283	-0.149908071
-0.268629129	-0.271358373	0.307246932	-0.317076926	0.195054931
-0.443451048	-0.31940573	0.267259046	-0.249286781	0.282159483
0.392436056	0.251855081	-0.302602447	0.279728672	-0.210358984
0.232463989	0.377392116	-0.27478475	0.249927179	-0.098777483

LV_syst_circumf_SAX_SR	LV_diast_radial_SAX_SR	LV_diast_circumf_SAX_SR	LV_radial_LAX_S
0.237180602	0.348822241	-0.328779288	-0.480328642
0.235053821	0.536384836	-0.507004999	-0.636539399
0.072953657	-0.139066273	0.13314694	0.060205218
-0.182295292	-0.567106667	0.511037117	0.569561085
0.180294643	0.276233665	-0.271908162	-0.430064545
0.219477957	0.525193834	-0.499586109	-0.630930239
-0.26479962	-0.769475333	0.651410159	0.693731489
0.249924136	0.739532397	-0.644138993	-0.73391632
-0.883006913	-0.4287553	0.361244035	0.374737899
1	0.149884265	-0.097718367	-0.187965083
0.149884265	1	-0.95130343	-0.599768186
-0.097718367	-0.95130343	1	0.528416436
-0.187965083	-0.599768186	0.528416436	1
0.072154484	9.5963E-05	-0.059608175	-0.189166629
-0.165289407	-0.521247683	0.464402992	0.629271189
0.194946053	0.592011826	-0.568340166	-0.638853696
0.11370146	0.744659093	-0.741428121	-0.762925966
-0.118525125	-0.670050748	0.698712187	0.633610191
0.184862481	0.182148603	-0.166758144	-0.271687147
0.131444092	0.381119284	-0.379203211	-0.448731855
0.115081387	-0.35151884	0.377307112	0.303032697
-0.090373055	-0.542822305	0.515831464	0.498543452
0.139416764	0.123113803	-0.125842908	-0.243749752
0.105106831	0.361548689	-0.364066546	-0.4258409
0.087516397	0.243291089	-0.234673912	-0.51489311
-0.123812136	-0.43611265	0.432244062	0.606046516
-0.010161992	0.169938548	-0.124514725	-0.156664997
-0.093866355	-0.374513692	0.357024949	0.388543049
-0.138243272	-0.270855527	0.276173661	0.343903096
0.150745157	0.481454963	-0.474017509	-0.501403316
0.077123322	0.243593355	-0.198929823	-0.253651429
-0.121274454	-0.236446028	0.174628119	0.421751051
0.007760967	0.27040201	-0.239458767	-0.172576773
-0.100262985	-0.247568305	0.197495762	0.319397004
-0.178712456	-0.334118449	0.340845072	0.237947571
0.137160873	0.242017577	-0.218756592	-0.288197992
-0.060340306	0.642758212	-0.685894102	-0.291352065

LV_time_to_peak_long_LAX	LV_syst_radial_LAX_SR	LV_syst_long_LAX_SR
0.238659968	-0.419196758	0.515770922
0.137882747	-0.511269301	0.594330623
0.201543854	-0.010632692	0.050843155
-0.009142377	0.473939457	-0.504962527
0.263628613	-0.348598217	0.424044453
0.136314368	-0.505092256	0.567576051
-0.023402323	0.477343727	-0.514834079
0.035284881	-0.454253828	0.511510582
-0.08249272	0.307578693	-0.366873049
0.072154484	-0.165289407	0.194946053
9.5963E-05	-0.521247683	0.592011826
-0.059608175	0.464402992	-0.568340166
-0.189166629	0.629271189	-0.638853696
1	-0.182316865	0.227244407
-0.182316865	1	-0.826502558
0.227244407	-0.826502558	1
0.083225657	-0.666179624	0.668843349
-0.131043379	0.704715609	-0.748273047
0.405667219	-0.288836392	0.374738424
0.41720722	-0.397389016	0.47980373
-0.000871662	0.178423876	-0.163924343
-0.111961875	0.416765958	-0.467585486
0.446863755	-0.223987023	0.292308855
0.402082602	-0.366448094	0.441816454
0.202497865	-0.376208851	0.390956505
-0.156237308	0.478572859	-0.540427191
-0.034123468	-0.21924697	0.162746603
-0.044547292	0.350574719	-0.340216037
-0.17340184	0.395913919	-0.43714463
0.24190028	-0.501137319	0.58244574
0.196903026	-0.257889599	0.310028101
-0.180218094	0.297810266	-0.346606725
0.110217274	-0.149755737	0.189588829
-0.198174608	0.212551664	-0.278472145
-0.283907193	0.300870973	-0.392363581
0.274229347	-0.267352258	0.316270347
-0.08744175	-0.455752751	0.484333713

LV_diast_radial_LAX_SR	LV_diast_long_LAX_SR	RV_EDV	RV_ESV	RV_SV
0.370970729	-0.379943328	0.678385389	0.65007194	0.090761338
0.533759561	-0.533422852	0.477136995	0.641708333	-0.267695967
-0.09886576	0.084077253	0.462359359	0.203086138	0.493131657
-0.495401729	0.488164383	-0.211355952	-0.473287482	0.462941502
0.276892973	-0.280056018	0.559436378	0.590541215	-0.033796757
0.514409874	-0.514369861	0.420659116	0.621885887	-0.341112995
-0.563373617	0.509486416	-0.183814616	-0.367249334	0.322598864
0.573654106	-0.507957878	0.175124846	0.377753592	-0.35865641
-0.349052545	0.317587311	-0.210575052	-0.217933224	-0.00413453
0.11370146	-0.118525125	0.184862481	0.131444092	0.115081387
0.744659093	-0.670050748	0.182148603	0.381119284	-0.35151884
-0.741428121	0.698712187	-0.166758144	-0.379203211	0.377307112
-0.762925966	0.633610191	-0.271687147	-0.448731855	0.303032697
0.083225657	-0.131043379	0.405667219	0.41720722	-0.000871662
-0.666179624	0.704715609	-0.288836392	-0.397389016	0.178423876
0.668843349	-0.748273047	0.374738424	0.47980373	-0.163924343
1	-0.837877722	0.257287263	0.405253566	-0.244721588
-0.837877722	1	-0.327665816	-0.430464336	0.154046044
0.257287263	-0.327665816	1	0.861353703	0.329263966
0.405253566	-0.430464336	0.861353703	1	-0.195491018
-0.244721588	0.154046044	0.329263966	-0.195491018	1
-0.44638849	0.423532738	-0.420659721	-0.777551768	0.634176299
0.200747837	-0.278921372	0.939499604	0.854452841	0.226424978
0.386691884	-0.410789972	0.813514692	0.970607624	-0.232548374
0.370322769	-0.290691493	0.189721843	0.364556314	-0.319756643
-0.532428129	0.434488551	-0.203326063	-0.380606478	0.317184803
0.063706534	-0.071627503	-0.016406519	0.150728725	-0.31456779
-0.364885983	0.320941747	-0.114798872	-0.370171286	0.465724066
-0.283853991	0.353750298	-0.199087999	-0.351732429	0.270000304
0.465205991	-0.43442778	0.302273441	0.432217411	-0.227490113
0.226937384	-0.214034468	0.324608861	0.387746324	-0.113293685
-0.352950089	0.375284485	-0.326586711	-0.335103651	-0.00370889
0.141207501	-0.171039667	0.200923487	0.337059809	-0.254604473
-0.288560858	0.268905164	-0.27605784	-0.310854577	0.051655986
-0.289180568	0.329300418	-0.360109718	-0.392403962	0.048367453
0.255849241	-0.320486026	0.386209802	0.354629039	0.076427573
0.51133913	-0.456996231	0.124402962	0.347187877	-0.407042485

RV_EF	RV_EDV/BSA	RV_ESV/BSA	LA_radial_LAX_S	LA_long_LAX_S
-0.473823427	0.579400295	0.590694897	0.382933904	-0.385458752
-0.668506272	0.413861357	0.603759458	0.396041338	-0.427714188
0.113124079	0.386611611	0.157621123	0.09489941	-0.057655564
0.706312552	-0.179246129	-0.465085129	-0.273855642	0.320668581
-0.457732418	0.558632497	0.567806977	0.417223621	-0.37567402
-0.687341061	0.408942789	0.605920205	0.404573679	-0.422040948
0.54345803	-0.152764825	-0.356853655	-0.283654119	0.372535947
-0.553300257	0.143279145	0.360510443	0.338545403	-0.413051832
0.245000688	-0.150446853	-0.186242752	-0.124889199	0.20280384
-0.090373055	0.139416764	0.105106831	0.087516397	-0.123812136
-0.542822305	0.123113803	0.361548689	0.243291089	-0.43611265
0.515831464	-0.125842908	-0.364066546	-0.234673912	0.432244062
0.498543452	-0.243749752	-0.4258409	-0.51489311	0.606046516
-0.111961875	0.446863755	0.402082602	0.202497865	-0.156237308
0.416765958	-0.223987023	-0.366448094	-0.376208851	0.478572859
-0.467585486	0.292308855	0.441816454	0.390956505	-0.540427191
-0.44638849	0.200747837	0.386691884	0.370322769	-0.532428129
0.423532738	-0.278921372	-0.410789972	-0.290691493	0.434488551
-0.420659721	0.939499604	0.813514692	0.189721843	-0.203326063
-0.777551768	0.854452841	0.970607624	0.364556314	-0.380606478
0.634176299	0.226424978	-0.232548374	-0.319756643	0.317184803
1	-0.411352764	-0.760964473	-0.380913919	0.414028503
-0.411352764	1	0.840350027	0.190642767	-0.175594525
-0.760964473	0.840350027	1	0.349887705	-0.35932193
-0.380913919	0.190642767	0.349887705	1	-0.904125147
0.414028503	-0.175594525	-0.35932193	-0.904125147	1
-0.313639917	-0.023632431	0.144854725	0.348712217	-0.318034152
0.550251118	-0.087414504	-0.355931693	-0.570408515	0.59101018
0.342693305	-0.225972598	-0.335745378	-0.74223804	0.702850442
-0.4069565	0.282470086	0.399202647	0.841518822	-0.911462652
-0.332748592	0.245171493	0.335064835	0.30302	-0.283299803
0.20896766	-0.272021444	-0.295595395	-0.321133605	0.348697643
-0.441090173	0.181244374	0.322062537	0.316273571	-0.276330026
0.263854439	-0.258456332	-0.301845212	-0.311918228	0.337075609
0.293060767	-0.312127846	-0.339115623	-0.306992198	0.34385884
-0.185414364	0.350934471	0.308012883	0.310065739	-0.312878383
-0.546403065	0.072596804	0.347711168	0.278933036	-0.415478789

LA_syst_radial_LAX_SR	LA_syst_long_LAX_SR	LA_diast_radial_LAX_SR	LA_diast_long_LAX_SR
0.143551038	-0.292219129	-0.304513329	0.461225419
0.213126523	-0.402167254	-0.344136087	0.417783906
-0.048428666	0.057286865	-0.03914468	0.191905718
-0.254458203	0.434243624	0.262147069	-0.279568213
0.15535754	-0.277224692	-0.326291053	0.46359118
0.219874691	-0.402029869	-0.357486224	0.416073571
-0.139116041	0.269947824	0.211335935	-0.372387995
0.113902686	-0.287036935	-0.259465874	0.398024929
-0.038823093	0.167974108	0.125944379	-0.259096338
-0.010161992	-0.093866355	-0.138243272	0.150745157
0.169938548	-0.374513692	-0.270855527	0.481454963
-0.124514725	0.357024949	0.276173661	-0.474017509
-0.156664997	0.388543049	0.343903096	-0.501403316
-0.034123468	-0.044547292	-0.17340184	0.24190028
-0.21924697	0.350574719	0.395913919	-0.501137319
0.162746603	-0.340216037	-0.43714463	0.58244574
0.063706534	-0.364885983	-0.283853991	0.465205991
-0.071627503	0.320941747	0.353750298	-0.43442778
-0.016406519	-0.114798872	-0.199087999	0.302273441
0.150728725	-0.370171286	-0.351732429	0.432217411
-0.31456779	0.465724066	0.270000304	-0.227490113
-0.313639917	0.550251118	0.342693305	-0.4069565
-0.023632431	-0.087414504	-0.225972598	0.282470086
0.144854725	-0.355931693	-0.335745378	0.399202647
0.348712217	-0.570408515	-0.74223804	0.841518822
-0.318034152	0.59101018	0.702850442	-0.911462652
1	-0.649179744	-0.338515453	0.29670282
-0.649179744	1	0.511168478	-0.532823354
-0.338515453	0.511168478	1	-0.741865297
0.29670282	-0.532823354	-0.741865297	1
0.0904796	-0.201180221	-0.154810454	0.325926285
0.093724923	0.09752537	0.230645767	-0.308789736
0.158753727	-0.390944039	-0.294361234	0.36139523
0.097098444	0.152452199	0.275472088	-0.377274043
-0.029363347	0.189289944	0.288966218	-0.437623698
-0.119995727	-0.061854311	-0.256942099	0.364821978
0.339807724	-0.50692256	-0.286260088	0.444924692

RA_radial_LAX_S	RA_long_LAX_S	RA_syst_radial_LAX_SR	RA_syst_long_LAX_SR
0.454876882	-0.323849634	0.316419532	-0.280204917
0.343086091	-0.313636533	0.296206083	-0.285388537
0.28023783	-0.110700433	0.120307728	-0.077719229
-0.213546913	0.204222107	-0.28698655	0.23920599
0.423650661	-0.254622791	0.344788347	-0.268629129
0.301804879	-0.269530503	0.305164712	-0.271358373
-0.233386227	0.345760586	-0.250674219	0.307246932
0.23525372	-0.342487875	0.270093283	-0.317076926
-0.171985375	0.180275223	-0.149908071	0.195054931
0.077123322	-0.121274454	0.007760967	-0.100262985
0.243593355	-0.236446028	0.27040201	-0.247568305
-0.198929823	0.174628119	-0.239458767	0.197495762
-0.253651429	0.421751051	-0.172576773	0.319397004
0.196903026	-0.180218094	0.110217274	-0.198174608
-0.257889599	0.297810266	-0.149755737	0.212551664
0.310028101	-0.346606725	0.189588829	-0.278472145
0.226937384	-0.352950089	0.141207501	-0.288560858
-0.214034468	0.375284485	-0.171039667	0.268905164
0.324608861	-0.326586711	0.200923487	-0.27605784
0.387746324	-0.335103651	0.337059809	-0.310854577
-0.113293685	-0.00370889	-0.254604473	0.051655986
-0.332748592	0.20896766	-0.441090173	0.263854439
0.245171493	-0.272021444	0.181244374	-0.258456332
0.335064835	-0.295595395	0.322062537	-0.301845212
0.30302	-0.321133605	0.316273571	-0.311918228
-0.283299803	0.348697643	-0.276330026	0.337075609
0.0904796	0.093724923	0.158753727	0.097098444
-0.201180221	0.09752537	-0.390944039	0.152452199
-0.154810454	0.230645767	-0.294361234	0.275472088
0.325926285	-0.308789736	0.36139523	-0.377274043
1	-0.677729912	0.388933276	-0.502357762
-0.677729912	1	-0.203109114	0.712188458
0.388933276	-0.203109114	1	-0.572445668
-0.502357762	0.712188458	-0.572445668	1
-0.724009625	0.596681268	-0.488055562	0.548787717
0.595906301	-0.773573983	0.348877459	-0.650188769
0.151891471	0.099309456	0.221694983	0.010125412

RA_diast_radial_LAX_SR	RA_diast_long_LAX_SR	Cardiac Sarcoidosis
-0.464287962	0.416398561	0.263812476
-0.348415211	0.280404686	0.373286581
-0.288228642	0.300795974	-0.062769007
0.18577694	-0.107521548	-0.432986577
-0.443451048	0.392436056	0.232463989
-0.31940573	0.251855081	0.377392116
0.267259046	-0.302602447	-0.27478475
-0.249286781	0.279728672	0.249927179
0.282159483	-0.210358984	-0.098777483
-0.178712456	0.137160873	-0.060340306
-0.334118449	0.242017577	0.642758212
0.340845072	-0.218756592	-0.685894102
0.237947571	-0.288197992	-0.291352065
-0.283907193	0.274229347	-0.08744175
0.300870973	-0.267352258	-0.455752751
-0.392363581	0.316270347	0.484333713
-0.289180568	0.255849241	0.51133913
0.329300418	-0.320486026	-0.456996231
-0.360109718	0.386209802	0.124402962
-0.392403962	0.354629039	0.347187877
0.048367453	0.076427573	-0.407042485
0.293060767	-0.185414364	-0.546403065
-0.312127846	0.350934471	0.072596804
-0.339115623	0.308012883	0.347711168
-0.306992198	0.310065739	0.278933036
0.34385884	-0.312878383	-0.415478789
-0.029363347	-0.119995727	0.339807724
0.189289944	-0.061854311	-0.50692256
0.288966218	-0.256942099	-0.286260088
-0.437623698	0.364821978	0.444924692
-0.724009625	0.595906301	0.151891471
0.596681268	-0.773573983	0.099309456
-0.488055562	0.348877459	0.221694983
0.548787717	-0.650188769	0.010125412
1	-0.798625367	-0.202985697
-0.798625367	1	-0.0140434
-0.202985697	-0.0140434	1
