# Supplementary material for: Machine-Learning-Based Diagnostics of Cardiac Sarcoidosis Using Multi-Chamber Wall Motion Analyses
Source: Diagnostics (Basel). 2023 Jul 20;13(14):2426. doi: 10.3390/diagnostics13142426 (PMC10377893; doi:10.3390/diagnostics13142426)
Supplement: Supplementary file 1 [file diagnostics-13-02426-s001.zip › S3_CMR+_CMR-_matrix.pdf]

	LV_EDV	LV_ESV	LV_SV	LV_EF	LV_EDV/BSA
LV_EDV	1	0.754636	0.571511	-0.32466	0.949450259
LV_ESV	0.75463585	1	-0.10704	-0.83398	0.687928584
LV_SV	0.571511306	-0.10704	1	0.550488	0.578305147
LV_EF	-0.324664656	-0.83398	0.550488	1	-0.287103951
LV_EDV/BSA	0.949450259	0.687929	0.578305	-0.2871	1
LV_ESV/BSA	0.729923231	0.981586	-0.121	-0.84182	0.718379008
LV_radial_SAX_S	-0.451277221	-0.74074	0.240841	0.7711	-0.408613547
LV_circumf_SAX_S	0.505560001	0.804141	-0.23808	-0.80273	0.453058884
LV_syst_radial_SAX_SR	-0.488570418	-0.73208	0.174308	0.699814	-0.456026928
LV_syst_circumf_SAX_SR	0.481028624	0.681991	-0.12413	-0.60002	0.466651911
LV_diast_radial_SAX_SR	0.398835207	0.654358	-0.21313	-0.61261	0.305757985
LV_diast_circumf_SAX_SR	-0.410467953	-0.60419	0.133386	0.490376	-0.314252472
LV_radial_LAX_S	-0.521697333	-0.66892	0.045838	0.554818	-0.455616695
LV_time_to_peak_long_LAX	0.340201045	0.204098	0.26069	-0.03967	0.352272852
LV_syst_radial_LAX_SR	-0.408982879	-0.53183	0.036017	0.459288	-0.363633722
LV_syst_long_LAX_SR	0.524230493	0.622452	0.015199	-0.46859	0.45659034
LV_diast_radial_LAX_SR	0.358322021	0.558804	-0.15264	-0.46465	0.246635611
LV_diast_long_LAX_SR	-0.356918078	-0.54514	0.137808	0.466398	-0.242854404
RV_EDV	0.613305108	0.425261	0.397779	-0.13757	0.519006356
RV_ESV	0.591501655	0.566477	0.187243	-0.36381	0.544408393
RV_SV	0.048439601	-0.2841	0.43058	0.46617	-0.062031478
RV_EF	-0.414112142	-0.6025	0.127724	0.622605	-0.42069934
RV_EDV/BSA	0.53596482	0.384111	0.332034	-0.14036	0.525995019
RV_ESV/BSA	0.540224907	0.529293	0.155978	-0.35824	0.523505553
LA_radial_LAX_S	0.556026319	0.489183	0.228324	-0.26509	0.575406997
LA_long_LAX_S	-0.454456938	-0.41635	-0.16508	0.207136	-0.438877595
LA_syst_radial_LAX_SR	0.096130085	0.141171	-0.03256	-0.17928	0.125678082
LA_syst_long_LAX_SR	-0.233928614	-0.31075	0.036591	0.325067	-0.241883151
LA_diast_radial_LAX_SR	-0.510951675	-0.4694	-0.18767	0.28679	-0.51886257
LA_diast_long_LAX_SR	0.529721943	0.363906	0.345405	-0.09565	0.543076577
RA_radial_LAX_S	0.451999943	0.310563	0.297028	-0.15511	0.419899087
RA_long_LAX_S	-0.379286422	-0.38627	-0.09374	0.268266	-0.306260735
RA_syst_radial_LAX_SR	0.347609128	0.265376	0.1952	-0.22564	0.370105816
RA_syst_long_LAX_SR	-0.337938472	-0.32866	-0.10301	0.262555	-0.312167316
RA_diast_radial_LAX_SR	-0.494274769	-0.31339	-0.35743	0.090151	-0.472545123
RA_diast_long_LAX_SR	0.460671753	0.303954	0.319294	-0.10133	0.4350224
Cardiac Sarcoidosis	0.374911098	0.361695	0.114767	-0.25437	0.318373162

LV_ESV/BSA	LV_radial_SAX_S	LV_circumf_SAX_S	LV_syst_radial_SAX_SR
0.729923231	-0.451277221	0.505560001	-0.488570418
0.981586	-0.740741009	0.804141236	-0.732082165
-0.12100188	0.240840629	-0.238077783	0.174308366
-0.841819628	0.771100221	-0.802734085	0.699813774
0.718379008	-0.408613547	0.453058884	-0.456026928
1	-0.751762684	0.806571818	-0.744622751
-0.751762684	1	-0.964852562	0.891873491
0.806571818	-0.964852562	1	-0.904310897
-0.744622751	0.891873491	-0.904310897	1
0.707622025	-0.795940898	0.805756224	-0.915843638
0.627649256	-0.815569869	0.802035325	-0.769201149
-0.57213879	0.65646008	-0.660318335	0.674451991
-0.656552288	0.755017166	-0.802434816	0.715470431
0.202247793	-0.019974805	0.048030169	-0.129631609
-0.541846712	0.648014208	-0.673968905	0.614330512
0.603130142	-0.658623735	0.696890278	-0.748650787
0.530175109	-0.634341333	0.670060877	-0.651892723
-0.520378384	0.606544648	-0.617217344	0.629868749
0.381665043	-0.150964988	0.162982522	-0.144184666
0.551915975	-0.336746094	0.361461073	-0.289001878
-0.349173327	0.384106967	-0.41116177	0.295995994
-0.629340638	0.558248209	-0.579409378	0.45177366
0.390704503	-0.14109246	0.140874784	-0.145260143
0.535408951	-0.325064708	0.336735728	-0.269247834
0.492157829	-0.428997118	0.512492891	-0.439122983
-0.404017603	0.4237824	-0.496718659	0.451185626
0.148687626	-0.096315604	0.094456019	-0.036803386
-0.312892418	0.23623567	-0.283910641	0.16990173
-0.483599845	0.403272356	-0.4794078	0.467231345
0.361782448	-0.312854836	0.388226161	-0.399319736
0.266358923	-0.227389633	0.236528454	-0.195330622
-0.34125144	0.431023383	-0.416736532	0.298916226
0.270660349	-0.174364704	0.21359307	-0.153438989
-0.308801998	0.312350276	-0.326134869	0.259247549
-0.280752946	0.224449604	-0.21859469	0.204650125
0.277237335	-0.315511785	0.291570753	-0.251709932
0.326568562	-0.355204376	0.379713679	-0.343962062

LV_syst_circumf_SAX_SR	LV_diast_radial_SAX_SR	LV_diast_circumf_SAX_SR	LV_radial_LAX_S
0.481028624	0.398835207	-0.410467953	-0.521697333
0.681990983	0.654358429	-0.604192278	-0.668922806
-0.124133462	-0.21313017	0.13338555	0.04583765
-0.600021115	-0.612614823	0.490376185	0.554818391
0.466651911	0.305757985	-0.314252472	-0.455616695
0.707622025	0.627649256	-0.57213879	-0.656552288
-0.795940898	-0.815569869	0.65646008	0.755017166
0.805756224	0.802035325	-0.660318335	-0.802434816
-0.915843638	-0.769201149	0.674451991	0.715470431
1	0.75441624	-0.7351585	-0.702275486
0.75441624	1	-0.935113434	-0.691771043
-0.7351585	-0.935113434	1	0.594482173
-0.702275486	-0.691771043	0.594482173	1
0.162342279	0.063382615	-0.219623529	-0.26120009
-0.581905303	-0.618593344	0.540510879	0.778555155
0.743664822	0.668691047	-0.642221926	-0.83630412
0.704464377	0.79850072	-0.779197779	-0.81512402
-0.708118468	-0.746568664	0.778187452	0.692016051
0.184829345	0.219364582	-0.287592858	-0.317735017
0.302719256	0.350596294	-0.381755938	-0.452084311
-0.234564465	-0.257792357	0.175909396	0.268453891
-0.396519126	-0.45010635	0.380006245	0.482350728
0.217951154	0.195389207	-0.267780604	-0.301267718
0.303370458	0.313348796	-0.336325154	-0.421085458
0.474213441	0.399756499	-0.362778045	-0.670273837
-0.507220287	-0.470862685	0.44324033	0.709385851
-0.019224505	-0.053999568	0.132067816	-0.111966838
-0.143908695	-0.158595847	0.08205721	0.340288832
-0.522441598	-0.490466188	0.484954806	0.645166433
0.475697595	0.405536135	-0.420462731	-0.603778031
0.042914227	0.251730432	-0.181077244	-0.235649344
-0.251319733	-0.495035977	0.437639512	0.518497006
0.065430734	0.114454473	-0.052926633	-0.152908575
-0.252667833	-0.370214996	0.319403904	0.412310766
-0.143277511	-0.321069183	0.354562465	0.265154035
0.219630046	0.378821983	-0.397330808	-0.327916316
0.357688042	0.396203313	-0.370481336	-0.477132071

LV_time_to_peak_long_LAX	LV_syst_radial_LAX_SR	LV_syst_long_LAX_SR
0.340201045	-0.408982879	0.524230493
0.204098369	-0.531828742	0.622451914
0.260689882	0.03601657	0.015198845
-0.039665157	0.459287506	-0.468588769
0.352272852	-0.363633722	0.45659034
0.202247793	-0.541846712	0.603130142
-0.019974805	0.648014208	-0.658623735
0.048030169	-0.673968905	0.696890278
-0.129631609	0.614330512	-0.748650787
0.162342279	-0.581905303	0.743664822
0.063382615	-0.618593344	0.668691047
-0.219623529	0.540510879	-0.642221926
-0.26120009	0.778555155	-0.83630412
1	-0.272358557	0.367281397
-0.272358557	1	-0.727723338
0.367281397	-0.727723338	1
0.192305848	-0.795560053	0.786494818
-0.219587602	0.691401377	-0.744948053
0.54493625	-0.304638199	0.402138856
0.542143285	-0.376728237	0.473896745
0.007820023	0.138772763	-0.131872414
-0.194841292	0.343159861	-0.399781581
0.564717206	-0.296760833	0.37776934
0.517083734	-0.353452919	0.445385148
0.337729093	-0.45943851	0.615184074
-0.26148335	0.488011654	-0.681945695
-0.035754056	-0.007207269	-0.069444744
-0.138786391	0.165589814	-0.162305496
-0.353683766	0.562537786	-0.642124945
0.38926002	-0.442051256	0.651114611
0.253819439	-0.243793049	0.324974679
-0.223263983	0.484861601	-0.515677713
0.122619625	-0.121789204	0.098828555
-0.208482256	0.392657581	-0.411272879
-0.331790794	0.286027456	-0.358630279
0.310074842	-0.322809085	0.378925769
0.22984484	-0.360585467	0.433967734

LV_diast_radial_LAX_SR	LV_diast_long_LAX_SR	RV_EDV	RV_ESV	RV_SV
0.358322021	-0.356918078	0.613305	0.591501655	0.048439601
0.558803505	-0.545144229	0.425261	0.566477127	-0.284097987
-0.152638767	0.137808242	0.397779	0.187243372	0.43057952
-0.464651617	0.466397645	-0.13757	-0.363814218	0.466169901
0.246635611	-0.242854404	0.519006	0.544408393	-0.062031478
0.530175109	-0.520378384	0.381665	0.551915975	-0.349173327
-0.634341333	0.606544648	-0.15096	-0.336746094	0.384106967
0.670060877	-0.617217344	0.162983	0.361461073	-0.41116177
-0.651892723	0.629868749	-0.14418	-0.289001878	0.295995994
0.704464377	-0.708118468	0.184829	0.302719256	-0.234564465
0.79850072	-0.746568664	0.219365	0.350596294	-0.257792357
-0.779197779	0.778187452	-0.28759	-0.381755938	0.175909396
-0.81512402	0.692016051	-0.31774	-0.452084311	0.268453891
0.192305848	-0.219587602	0.544936	0.542143285	0.007820023
-0.795560053	0.691401377	-0.30464	-0.376728237	0.138772763
0.786494818	-0.744948053	0.402139	0.473896745	-0.131872414
1	-0.879178066	0.328577	0.396909718	-0.112832229
-0.879178066	1	-0.39451	-0.430338108	0.040832369
0.328576731	-0.394514156	1	0.891931809	0.257608838
0.396909718	-0.430338108	0.891932	1	-0.206322098
-0.112832229	0.040832369	0.257609	-0.206322098	1
-0.342531114	0.355599657	-0.42252	-0.746544997	0.682073491
0.301872801	-0.371296565	0.947194	0.892965276	0.142390561
0.374585772	-0.408943658	0.854993	0.967897332	-0.216783094
0.476536436	-0.38696319	0.348414	0.480288095	-0.287517037
-0.60200696	0.487038774	-0.30059	-0.38940251	0.184863264
-0.09524752	0.07624394	-0.09625	0.057560506	-0.33557365
-0.166694933	0.162126005	-0.09175	-0.307210872	0.455381088
-0.588629317	0.5512515	-0.42755	-0.533235003	0.215082442
0.506773565	-0.430158427	0.400565	0.446428159	-0.100234599
0.180352376	-0.134814179	0.313903	0.368817941	-0.134796372
-0.545656344	0.518143304	-0.34991	-0.407669323	0.119885308
0.001023495	-0.071104448	0.237884	0.340204974	-0.2363839
-0.440881087	0.373789816	-0.32345	-0.363348121	0.086751703
-0.261649291	0.293240429	-0.4034	-0.396228688	-0.005755253
0.322155067	-0.391449306	0.412462	0.397245812	0.029750414
0.425272271	-0.384378668	0.219067	0.258844599	-0.071657498

RV_EF	RV_EDV/BSA	RV_ESV/BSA	LA_radial_LAX_S	LA_long_LAX_S
-0.414112142	0.53596482	0.540224907	0.556026319	-0.454456938
-0.602500248	0.384110547	0.529292945	0.489182883	-0.416352543
0.127723991	0.332034169	0.155978324	0.228324051	-0.16508253
0.622605382	-0.140359856	-0.35823939	-0.265094065	0.207135884
-0.42069934	0.525995019	0.523505553	0.575406997	-0.438877595
-0.629340638	0.390704503	0.535408951	0.492157829	-0.404017603
0.558248209	-0.14109246	-0.325064708	-0.428997118	0.4237824
-0.579409378	0.140874784	0.336735728	0.512492891	-0.496718659
0.45177366	-0.145260143	-0.269247834	-0.439122983	0.451185626
-0.396519126	0.217951154	0.303370458	0.474213441	-0.507220287
-0.45010635	0.195389207	0.313348796	0.399756499	-0.470862685
0.380006245	-0.267780604	-0.336325154	-0.362778045	0.44324033
0.482350728	-0.301267718	-0.421085458	-0.670273837	0.709385851
-0.194841292	0.564717206	0.517083734	0.337729093	-0.26148335
0.343159861	-0.296760833	-0.353452919	-0.45943851	0.488011654
-0.399781581	0.37776934	0.445385148	0.615184074	-0.681945695
-0.342531114	0.301872801	0.374585772	0.476536436	-0.60200696
0.355599657	-0.371296565	-0.408943658	-0.38696319	0.487038774
-0.422523912	0.94719399	0.854993294	0.348413626	-0.30059072
-0.746544997	0.892965276	0.967897332	0.480288095	-0.38940251
0.682073491	0.142390561	-0.216783094	-0.287517037	0.184863264
1	-0.447771376	-0.722070348	-0.461693284	0.336022753
-0.447771376	1	0.881764611	0.347790465	-0.289866021
-0.722070348	0.881764611	1	0.447731318	-0.355908195
-0.461693284	0.347790465	0.447731318	1	-0.935199169
0.336022753	-0.289866021	-0.355908195	-0.935199169	1
-0.248956399	-0.076166867	0.045837302	0.170336622	-0.066050714
0.489983316	-0.112280324	-0.291478473	-0.408328114	0.326876866
0.474424356	-0.47277344	-0.496510118	-0.815454057	0.813339484
-0.313214742	0.408226116	0.398983059	0.902115243	-0.927297702
-0.31243581	0.235919753	0.311032214	0.398455493	-0.290241239
0.327707246	-0.290759673	-0.367644587	-0.535538411	0.52566193
-0.460832775	0.210869575	0.31361979	0.383640847	-0.215702177
0.336091466	-0.289735782	-0.349408112	-0.49638034	0.456305547
0.257054928	-0.358468184	-0.333370079	-0.42368339	0.372984686
-0.242655222	0.374594081	0.34916088	0.460422578	-0.424320748
-0.212199842	0.179806131	0.279320039	0.340755495	-0.402859677

LA_syst_radial_LAX_SR	LA_syst_long_LAX_SR	LA_diast_radial_LAX_SR	LA_diast_long_LAX_SR
0.096130085	-0.233928614	-0.510951675	0.529721943
0.141170586	-0.310747821	-0.469400512	0.363905567
-0.032559608	0.036591128	-0.187667892	0.34540505
-0.179280022	0.325067363	0.2867896	-0.095646143
0.125678082	-0.241883151	-0.51886257	0.543076577
0.148687626	-0.312892418	-0.483599845	0.361782448
-0.096315604	0.23623567	0.403272356	-0.312854836
0.094456019	-0.283910641	-0.4794078	0.388226161
-0.036803386	0.16990173	0.467231345	-0.399319736
-0.019224505	-0.143908695	-0.522441598	0.475697595
-0.053999568	-0.158595847	-0.490466188	0.405536135
0.132067816	0.08205721	0.484954806	-0.420462731
-0.111966838	0.340288832	0.645166433	-0.603778031
-0.035754056	-0.138786391	-0.353683766	0.38926002
-0.007207269	0.165589814	0.562537786	-0.442051256
-0.069444744	-0.162305496	-0.642124945	0.651114611
-0.09524752	-0.166694933	-0.588629317	0.506773565
0.07624394	0.162126005	0.5512515	-0.430158427
-0.096245415	-0.091747225	-0.42754575	0.400565193
0.057560506	-0.307210872	-0.533235003	0.446428159
-0.33557365	0.455381088	0.215082442	-0.100234599
-0.248956399	0.489983316	0.474424356	-0.313214742
-0.076166867	-0.112280324	-0.47277344	0.408226116
0.045837302	-0.291478473	-0.496510118	0.398983059
0.170336622	-0.408328114	-0.815454057	0.902115243
-0.066050714	0.326876866	0.813339484	-0.927297702
1	-0.763475651	-0.183678112	-0.019748328
-0.763475651	1	0.43094736	-0.23975899
-0.183678112	0.43094736	1	-0.859153429
-0.019748328	-0.23975899	-0.859153429	1
0.083149122	-0.139909244	-0.236607093	0.344142497
0.059476813	0.156327413	0.435226628	-0.484600179
0.22297934	-0.377064941	-0.372736315	0.300670854
0.100261306	0.179793155	0.459214275	-0.493903815
0.04374263	0.071049323	0.346084962	-0.432791247
-0.144788673	-0.061242644	-0.378397069	0.475109157
0.227657381	-0.3863537	-0.345470772	0.314946612

RA_radial_LAX_S	RA_long_LAX_S	RA_syst_radial_LAX_SR	RA_syst_long_LAX_SR
0.451999943	-0.379286422	0.347609128	-0.337938472
0.310562884	-0.386266281	0.265375915	-0.328664206
0.297028176	-0.093736889	0.195199949	-0.103009845
-0.155109287	0.26826644	-0.2256426	0.262555074
0.419899087	-0.306260735	0.370105816	-0.312167316
0.266358923	-0.34125144	0.270660349	-0.308801998
-0.227389633	0.431023383	-0.174364704	0.312350276
0.236528454	-0.416736532	0.21359307	-0.326134869
-0.195330622	0.298916226	-0.153438989	0.259247549
0.042914227	-0.251319733	0.065430734	-0.252667833
0.251730432	-0.495035977	0.114454473	-0.370214996
-0.181077244	0.437639512	-0.052926633	0.319403904
-0.235649344	0.518497006	-0.152908575	0.412310766
0.253819439	-0.223263983	0.122619625	-0.208482256
-0.243793049	0.484861601	-0.121789204	0.392657581
0.324974679	-0.515677713	0.098828555	-0.411272879
0.180352376	-0.545656344	0.001023495	-0.440881087
-0.134814179	0.518143304	-0.071104448	0.373789816
0.313902769	-0.349908939	0.237884154	-0.323448223
0.368817941	-0.407669323	0.340204974	-0.363348121
-0.134796372	0.119885308	-0.2363839	0.086751703
-0.31243581	0.327707246	-0.460832775	0.336091466
0.235919753	-0.290759673	0.210869575	-0.289735782
0.311032214	-0.367644587	0.31361979	-0.349408112
0.398455493	-0.535538411	0.383640847	-0.49638034
-0.290241239	0.52566193	-0.215702177	0.456305547
0.083149122	0.059476813	0.22297934	0.100261306
-0.139909244	0.156327413	-0.377064941	0.179793155
-0.236607093	0.435226628	-0.372736315	0.459214275
0.344142497	-0.484600179	0.300670854	-0.493903815
1	-0.707694574	0.41072517	-0.548278771
-0.707694574	1	-0.229668368	0.757451667
0.41072517	-0.229668368	1	-0.507194538
-0.548278771	0.757451667	-0.507194538	1
-0.753258796	0.67551981	-0.457579503	0.577922722
0.614360475	-0.786197526	0.374708981	-0.696126851
0.219251082	-0.361663422	0.151634645	-0.405053905

RA_diast_radial_LAX_SR	RA_diast_long_LAX_SR	Cardiac Sarcoidosis
-0.494274769	0.460671753	0.374911098
-0.313393854	0.303954436	0.361694984
-0.357429705	0.319293942	0.114767132
0.090150857	-0.101328039	-0.254371997
-0.472545123	0.4350224	0.318373162
-0.280752946	0.277237335	0.326568562
0.224449604	-0.315511785	-0.355204376
-0.21859469	0.291570753	0.379713679
0.204650125	-0.251709932	-0.343962062
-0.143277511	0.219630046	0.357688042
-0.321069183	0.378821983	0.396203313
0.354562465	-0.397330808	-0.370481336
0.265154035	-0.327916316	-0.477132071
-0.331790794	0.310074842	0.22984484
0.286027456	-0.322809085	-0.360585467
-0.358630279	0.378925769	0.433967734
-0.261649291	0.322155067	0.425272271
0.293240429	-0.391449306	-0.384378668
-0.403395811	0.412461576	0.219066538
-0.396228688	0.397245812	0.258844599
-0.005755253	0.029750414	-0.071657498
0.257054928	-0.242655222	-0.212199842
-0.358468184	0.374594081	0.179806131
-0.333370079	0.34916088	0.279320039
-0.42368339	0.460422578	0.340755495
0.372984686	-0.424320748	-0.402859677
0.04374263	-0.144788673	0.227657381
0.071049323	-0.061242644	-0.3863537
0.346084962	-0.378397069	-0.345470772
-0.432791247	0.475109157	0.314946612
-0.753258796	0.614360475	0.219251082
0.67551981	-0.786197526	-0.361663422
-0.457579503	0.374708981	0.151634645
0.577922722	-0.696126851	-0.405053905
1	-0.851165857	-0.320701358
-0.851165857	1	0.339924347
-0.320701358	0.339924347	1
